# Hemiparesis post cerebral malaria

**DOI:** 10.11604/pamj.2015.20.1.5568

**Published:** 2015-01-01

**Authors:** Oumkaltoum Taiaa, Touriya Amil, Abdelatif Darbi

**Affiliations:** 1 Department of Radiology, Military Hospital Mohamed V Instruction, Rabat, Morocco

**Keywords:** Brain, plasmodium falciparum, magnetic resonance imaging, cerebral malaria

## Abstract

Cerebral malaria is one of the most serious complications in the Plasmodium falciparum infection. In endemic areas, the cerebral malaria interested mainly children. The occurrence in adults is very rare and most interested adult traveling in tropical zones. This case report describes a motor deficit post cerebral malaria in a young adult traveling in malaria endemic area. This complication has been reported especially in children and seems very rare in adults.

## Introduction

Cerebral malaria due to Plasmodium falciparum infection is a major cause of morbidity and mortality in the developing world [1, 2]. Unrouseable coma in patients with severe malaria is caused by diffuse symmetrical encephalopathy associated with the sequestration of erythrocytes containing mature stages of the parasite in the cerebral microvasculature [2]. The mortality rate associated with this disease is 15%-20%. In many respects, cerebral malaria is similar to a metabolic encephalopathy of short duration, with the majority of survivors making full neurological recoveries [3, 4]. However, 10% of children and a smaller proportion of adults with cerebral malaria [3] will have residual neurological sequel following recovery of consciousness. There are in the literature relatively few data regarding the in vivo imaging studies of cerebral malaria [2–4] and only one with hemiparesis post cerebral malaria [5].

## Patient and observation

**Figure 1:**
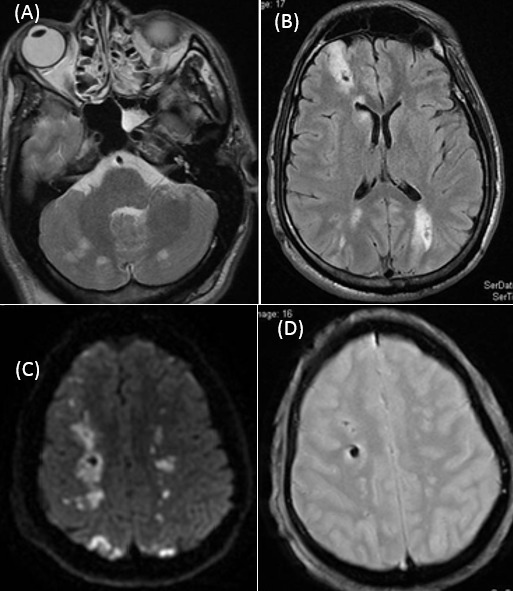
(A, B, C) white matter changes. The first examination, 3 days after the onset of cerebral malaria, shows diffuses hyperintensities on T2, T2- FLAIR-weighted and the SWI sequences images in the centrum semiovale, periventricular, frontal and occipital white matter, and in the cerebellum; (D) with central microhemorrhages in the occipital, frontal and right semiovale centrum white matter lesions on Gradient Echo (T2*) sequence

A 32-year-old Moroccan traveling in the Democratic Republic of Congo for five month, had neglected his malaria chemoprophylaxis. He developed fever, confusion and became rapidly comatose. So he was transferred to Morocco. At neurologic examination, Glasgow Coma Scale (GCS) was at 7, and he experienced in the emergency department an episode of tonic-clonic seizures lasting 30 seconds that was controlled by phenytoin administration. Plasmodium falciparum was seen on the blood smear. Other complications of malaria included acute renal failure, hepatic failure and retinopathy was determined. The patient was placed on mechanical ventilation. Specific treatment was started with intravenous doxycycline and quinine dihydrochloride. A brain CT on the first day was normal. A first brain magnetic resonance imaging (MRI), on day 3, performed on a 1.5-T unit included sagittal and axial 5-mm-thick T1-weighted images, axial 4-mm-thick T2-weighted images, susceptibility-weighted imaging(SWI), Gradient Echo (T2*) sequence, axial 4-mm-thick T1-weighted images, before and after contrast injection, and axial fluid-attenuated inversion-recovery (FLAIR) sequences. The MRI showed diffuses hyperintensities on T2, T2- FLAIR-weighted and the SWI sequences images in the centrum semiovale, periventricular, frontal and occipital white matter, and in the cerebellum (Figure 1A, B, C) with central micro hemorrhages in the occipital, frontal and right semiovale centrum white matter lesions on Gradient Echo (T2*) sequence (Figure 1D). There was no enhancement on T1-weighted images. There was also no evidence for dural sinus thrombosis.

**Figure 2:**
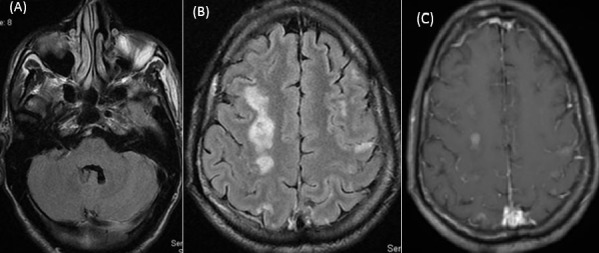
(A,B) at follow-up examination, Two week after the onset of illness. In comparison with the first examination, T2-FLAIR images show resolution of cerebellum hyperintensities,and minimal decrease in the size of the lesion of occipital, frontal and right semiovale centrum white matter; (C) the unenhanced microhaemorrhagic lesionson contrast-enhanced T1-weighted images

The GCS slightly improved up to 9. Two weeks after the onset of neurologic symptoms, He was progressively weaned from the mechanical ventilation. There was also a recovery of renal and hepatic function. Two weeks after the first examination (day 15), MRI showed disappearance of the cerebellum and periventricular lesions and minimal decrease in the size of the lesion of occipital, frontal and right semiovale centrum white matter (Figure 2A, B). There was a few central enhancement of white matter haemorrhagic lesions on post-contrast T1-weigted images (Figure 2C). On day 32, the patient regained consciousness, had been discharged to a rehabilitation unit. After a 4 months follow-up, no significant neurological improvement was noted and the patient was classified left hemiparesis post cerebral malaria.

## Discussion

Patients infected with the malaria parasite Plasmodium falciparum may develop a diffuse reversible encephalopathy, termed cerebral malaria. The neurologic signs, caused by diffuse involvement of the brain, are usually nonspecific [5]. It is unclear how the intraerythrocytic parasite, which sequesters in the cerebral microvasculature but does not enter the brain parenchyma, induces this neurological syndrome [1, 2, 4, 5]. In comatose patients, it is possible to observe brain edema on brain CT scan, but usually no significant change is apparent [5]. As falciparum malaria is characterized by a hypercoagulable state, cerebral venous and dural sinus thrombosis are possible complications that could be missed on brain CT scan [6]. Microhemorrhages can be seen in the cerebral white matter but not in the gray matter and in both gray and white matter in the cerebellum [4].

There are few reports of MRI findings in cerebral malaria [5, 6]. In a prospective MR study of 24 patients with cerebral malaria imaged on a 0.2-T confirmed that apart from a moderate brain swelling, MR imaging revealed no abnormalities [3]. Other [6, 7], reported ischemic changes occur in cerebral grey matter; the more obvious lesions are found around the veins in the white matter. The hemorrhages, microglial nodules and areas of necrosis and edema in the white matter in cerebral malaria closely resemble the white matter lesions in fat embolism [7]. Focal infarcts were also found in the basal ganglia, thalamus, pons, and cerebellum [6]. Cortical infarctions have a low specificity particularly in elderly patients. The other hypothesis in according with the possible pathogenesis of cerebral malaria is an ischemic injury do to the sequestration of infected erythrocytes in brain capillary [7].

Our patients, with treatment, regained consciousness, left hemiparesis was determined. A follow-up examination showed the disappearance of the white matter hyperintensities; periventricular and in the cerebellum probably do to cytotoxic edema [5, 6] and minimal decrease in the size of the micro hemorrhagic lesions of the right centrum semiovale, probably related to irreversible necrotic and hemorrhagic lesions of the perivascular myelin, which have been found to be similar to lesions caused by fat emboli [5, 7] which might explain the persistence of hemiparesis in our case.

## Conclusion

Central nervous system (CNS) complications occur in 2% of patients with acute malaria. These include cerebral edema, infarcts, and petechial hemorrhages. Our observations illustrate two kinds of cerebral lesions; reversible cytotoxic edema and irreversible necrotic and hemorrhagic lesions which might explain the persistence of hemiparesis.
